# Cadmium May Affect Newborn Girls More than Boys: Maternal Exposure Linked to Smaller Birth Size

**DOI:** 10.1289/ehp.120-a76b

**Published:** 2012-02-01

**Authors:** Julia R. Barrett

**Affiliations:** Julia R. Barrett, MS, ELS, a Madison, WI–based science writer and editor, has written for *EHP* since 1996. She is a member of the National Association of Science Writers and the Board of Editors in the Life Sciences.

Chronic exposure to cadmium, which primarily occurs through diet and smoking, damages the kidneys, weakens bones, and may increase cancer risk. The metal is also an endocrine disrupter and may adversely affect reproduction and child development. Animal studies indicate cadmium induces multiple defects in developing embryos, but it is unknown whether similar effects occur in humans. However, a new study shows that maternal cadmium exposure is associated with reduced head circumference and birth weight in newborn girls [*EHP* 120(2):284–289; Kippler et al.].

The study used data collected from more than 1,600 pregnant women participating in the Maternal and Infant Nutrition Interventions, a food and micronutrient supplementation trial in Matlab, Bangladesh. The women provided urine samples and completed clinical examinations in early pregnancy. Information on demographics and tobacco and betel use during pregnancy also was collected, along with newborns’ sex and physical measurements.

Average adult daily intakes of cadmium were estimated on the basis of rice consumption to be 25–35 µg, similar to those in other Asian countries where rice is a staple but higher than levels reported in U.S. and European women. Statistical analyses, controlled for confounding by maternal, birth, and newborn factors, suggested that for each 1-µg/L increase in maternal urinary cadmium, head circumference decreased by 0.26 cm and weight dropped by 45 g on average among newborn girls. Far smaller associations were seen for boys.

Cadmium may affect fetal growth by several mechanisms, including impaired nutrient transport and endocrine disruption. Sex-based differences in specific hormone levels as well as in cadmium uptake and mechanisms of action—which have been reported in experimental studies—may have played a role in the current findings. The authors say the estimated average reductions in head circumference and weight are unlikely to be clinically significant for individual children but could have public health implications given widespread dietary cadmium exposure and adverse effects associated with reduced birth size.

The main strengths of the study are its prospective design, large number of participants, wide range of exposure, and consideration of numerous confounding factors. However, the potential for unmeasured or residual confounding remained, as in all observational studies, and infants’ cadmium exposure was not directly assessed (for instance, by measuring cadmium levels in cord blood). Continuing research should follow the children to determine if growth effects are sustained through childhood and whether other health changes appear later in childhood.

**Figure:**
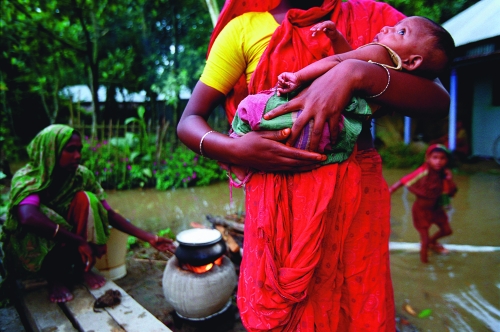
Asian diets high in rice consumption may lead to elevated cadmium intake, which has been linked to reduced birth size in girls. © 2012 G.M.B. Akash/Panos Pictures

